# A case of unusual renal manifestation in a patient with neuronal intranuclear inclusion disease treated with steroids

**DOI:** 10.1002/ccr3.7730

**Published:** 2023-08-08

**Authors:** Keisuke Morita, Takahiro Shinzato, Yuzo Endo, Makoto Suzuki, Hidefumi Yoshida, Jun Sone, Kojiro Nagai

**Affiliations:** ^1^ Department of Nephrology Shizuoka General Hospital Shizuoka Japan; ^2^ Department of Diagnostic Pathology Shizuoka General Hospital Shizuoka Japan; ^3^ Department of Neurology Shizuoka General Hospital Shizuoka Japan; ^4^ Department of Neuropathology, Institute for Medical Science of Aging Aichi Medical University Aichi Japan

**Keywords:** crescentic nephritis, fibroblast, IgA nephropathy, neuronal intranuclear inclusion disease, steroids

## Abstract

Neuronal intranuclear inclusion disease (NIID) is a progressive neurodegenerative disorder characterized by intranuclear inclusions. Kidney injury involvement and successful treatment for NIID have rarely been reported. A NIID patient developed crescentic IgA nephropathy. Steroid therapy resolved digestive symptoms and recovered renal function. Steroids are considered for concomitant symptoms of NIID.

## Graphical abstract

(A,B) Renal biopsy specimen showed numerous cellular to fibrocellular crescents (C, D) Immunofluorescence staining of IgA (C) and C3 (D). (E‐G) Merge images of the kidney immunostained by anti‐p62 antibody (red), ubiquitin antibody (green) and nuclear antibody (blue). Renal interstitial fibroblasts were positive for immunofluorescence staining, indicating intranuclear inclusion lesions (arrow).

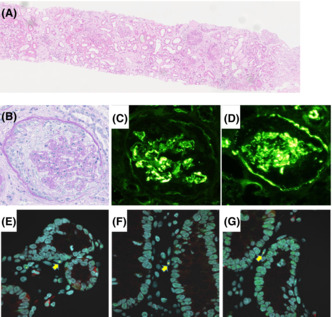

## 1 INTRODUCTION

Neuronal intranuclear inclusion disease (NIID) is a progressive neurodegenerative disorder characterized by intranuclear inclusions in various cells including central and peripheral nervous system, visceral organs, and skin.^1^ Patients with NIID present with wide variety of symptoms such as dementia, leukoencephalopathy, acute encephalitis‐like episodes, parkinsonism, essential tremor, peripheral neuropathy.^2^, ^3^, ^4^, ^5^, ^6^ Brain magnetic resonance imaging (MRI) findings of high‐intensity signal in the corticomedullary junction on diffusion‐weighted imaging is specific for NIID,^2^ and it is diagnosed pathologically by skin biopsy showing eosinophilic hyaline intranuclear inclusions^7^ and genetically by the expansion of the CGG repeats in the NOTCH2NLC gene.^8^ Although intranuclear inclusion lesions are also positive in multiple visceral organ cells in NIID patients,^1^, ^2^ involvement of other organ dysfunction such as kidney injury has rarely been reported.

We herein report a rare case of NIID patient complicated by rapid progressive glomerulonephritis with nephrotic‐range proteinuria. Renal biopsy revealed crescentic IgA nephropathy and intranuclear inclusion bodies in the interstitial fibroblasts. Kidney dysfunction and severe encephalitis‐like symptoms, which have presumably developed at the same time, were successfully treated with steroid therapy.

## 2 CASE PRESENTATION

We present a 73‐year‐old female with dementia and loss of motivation, such as closing her self‐employed store before the advertised time, which her family members began to notice 3 years ago. The serum creatinine was 0.68 mg/dL 7 years ago, and urinalysis had never been performed. She was admitted to the previous hospital (Day −41) for a lumbar compression fracture. Her serum creatinine was 1.2 mg/dL and urinalysis was not performed. She was making good progress in rehabilitation, but 1 month after admission (Day −10), she suddenly developed persistent nausea and vomiting, and her serum creatinine elevated to 2.33 mg/dL with massive hematuria and proteinuria. She was transferred to our hospital for further examination. She had a history of hypertension. She had no family history of neurological or renal dysfunction. Her activities of daily living were independent before hospital admission. She had received denosumab for osteoporosis a month before, and her regular medication included amlodipine, allopurinol, calcium carbonate, cholecarciferol, and magnesium carbonate.

**FIGURE 1:**
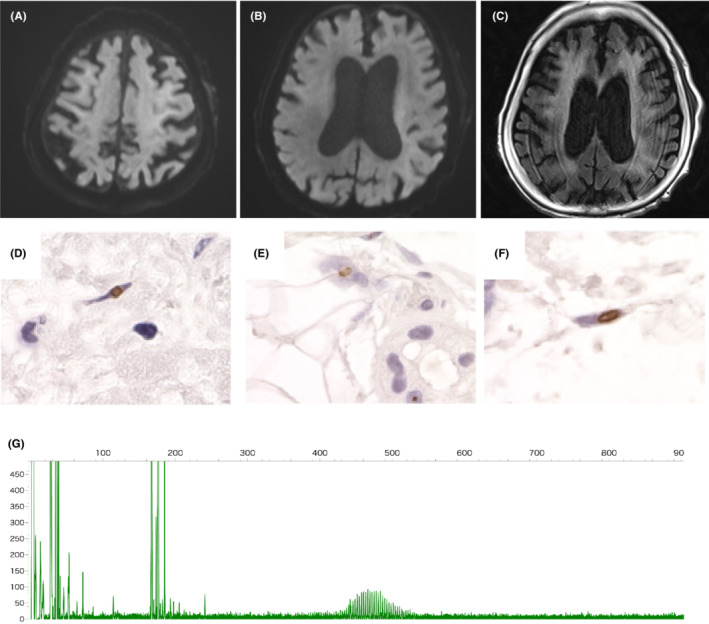
Diagnostic pictures. (A–C) Brain magnetic resonance imaging demonstrated high intensity signal in the corticomedullary junction on diffusion‐weighted imaging (DWI) (A,B) and sporadic high intensity lesions in the white matter of frontoparietal lobe on T2‐weighted fluid‐attenuated inversion recovery (FLAIR) (C). (D–F) Skin biopsy revealed that intranuclear inclusions found in the fibroblasts (D), sweat gland cells (E), and adipocytes (F) were positive for immunofluorescence staining with anti‐p62 antibody. (G) Fluorescent amplicon length analysis revealed CGG repeat expansion in the NOTCH2NLC gene.

**FIGURE 2:**
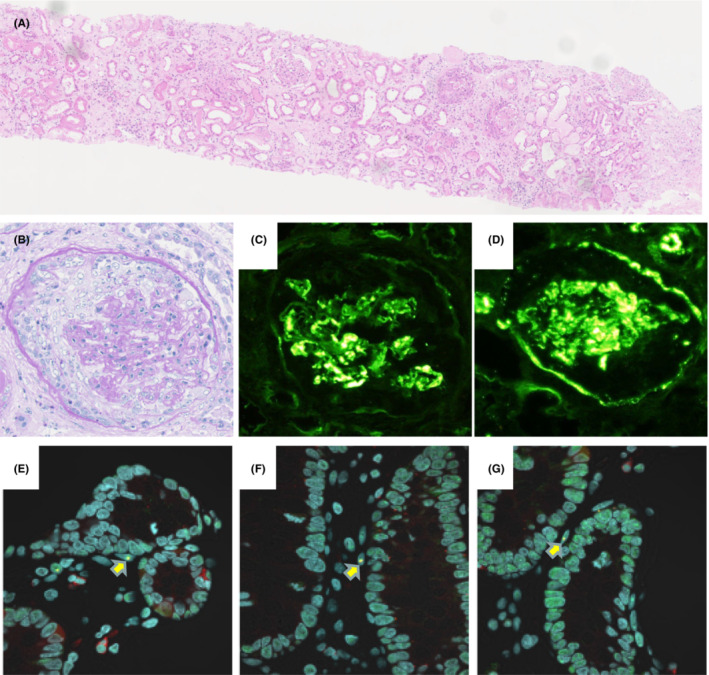
Representative pictures obtained from renal biopsy. (A,B) Renal biopsy specimen showed numerous cellular to fibrocellular crescents (A: hematoxylin and eosin stain, ×50. B: periodic acid‐shiff stain, ×200). (C, D) Immunofluorescence staining revealed granular mesangial deposition of. IgA (C) and C3 (D). (E–G) Merge images of the kidney immunostained by anti‐p62 antibody (red), ubiquitin antibody (green) and nuclear antibody (blue). Renal interstitial fibroblasts were positive for immunofluorescence staining with anti‐p62 antibody and ubiquitin antibody, indicating intranuclear inclusion lesions (arrow), whereas kidney‐specific cells such as tubular epithelial cells, podocytes or glomerular endothelial cells were not stained.

On physical examination, her body temperature was 36.6°C, blood pressure was 168/88 mmHg, heart rate was 70/min with a regular rhythm. Glasgow Coma Scale was 13 (E2V5M6), and Japan Coma Scale was 30. Physical examination revealed no abnormalities except for severe nausea, vomiting, and pretibial edema. Neurological examination revealed consciousness disturbance, symmetrical muscle weakness and sensory disturbances in the lower limbs, and bladder dysfunction. Blood test results were: white blood cells 12.6 × 10^9^ cells/L, hemoglobin 8.8 g/dL, total protein 5.1 g/dL, albumin 2.6 g/dL, creatinine 3.19 mg/dL, C‐reactive protein 1.73 mg/dL, IgG 432 mg/dL, IgA 323 mg/dL, IgM 30 mg/dL. Urinalysis revealed massive hematuria (50‐99/HPF) and proteinuria (29.38 g/gCr). Serological findings including antinuclear antibody, MPO‐ANCA, PR3‐ANCA, anti‐glomerular basement membrane antibody were all negative (Table 1). Mini‐Mental State Examination and Frontal Assessment Battery score were 26/30 and 13/18, respectively. Brain MRI revealed high intensity signal in the corticomedullary junction on diffusion‐weighted imaging (Figure 1A,B) and sporadic high intensity lesions in the white matter (Figure 1C). Cerebrospinal fluid examination could not be performed due to continual nausea and vomiting. A nerve conduction study revealed peripheral motor and sensory axonal neuropathy. Skin biopsy revealed p62‐positive intranuclear inclusions in the fibroblasts, sweat gland cells and adipocytes (Figure 1D–F). Fluorescent amplicon length analysis^8^ revealed CGG repeat expansion in the NOTCH2NLC gene (the length of GGC repeat expansion: 93) (Figure 1G), which was compatible with NIID. Renal biopsy was performed, which revealed numerous cellular to fibrocellular crescents (19/36 glomeruli) (Figure 2A,B). Immunofluorescence microscopy revealed granular IgA and C3 mesangial deposition (Figure 2C,D). Interstitial fibroblasts were positive for anti‐p62 immunofluorescence staining, whereas kidney‐specific cells such as tubular cells, podocytes or endothelial cells were negative (Figure 2E–G). Immunohistochemical staining for SV40, which is often utilized for diagnosis of virus nephropathy, was negative (data not shown). Following these findings, the patient was diagnosed with crescentic IgA nephropathy concurrent with neurological symptoms induced by NIID.

**TABLE 1:** Laboratory findings.

Blood count			Immunology		
White blood cell	12,600	×10^3^/μL	Immunoglobulin G	432	mg/dL
Neutrophil	75.1	%	Immunoglobulin A	323	mg/dL
Lymphocyte	12.4	%	Immunoglobulin M	30	mg/dL
Red blood cell	2.89	×10^6^/μL	Complement 3	90.0	mg/dL
Hemoglobin	8.8	g/dL	Complement 4	31.4	mg/dL
Hematocrit	25.8	%	CH50	60	U/mL
Platelet	462	×10^3^/μL	Rheumatoid factor	4.4	IU/mL
Biochemistry			Antinuclear antibody	<40	
Total protein	5.1	g/dL	Anti‐DNA antibody	<1.7	IU/mL
Albumin	2.6	g/dL	Anti‐GBM antibody	<2.0	U/mL
Urinary acid	5.4	mg/dL	MPO‐ANCA	0.8	U/mL
Blood urea nitrogen	39	mg/dL	PR3‐ANCA	<0.6	U/mL
Creatinine	3.19	mg/dL	Urinalysis		
Aspartate aminotransferase	28	IU/L	Protein	4+	
Alanine aminotransferase	17	IU/L	Occult blood	3+	
Alkaline phosphatase	99	IU/L	Red blood cell	>100	/HPF
Lactate dehydrogenase	247	IU/L	Protein content	29.38	g/gCre
Total bilirubin	0.4	mg/dL	beta‐2‐microglobulin	65,200	μg/L
Sodium	132	mEq/L	Bence‐Jones protein	(−)	
Potassium	3.5	mEq/L	–	–	–
Chloride	98	mEq/L	–	–	–
Calcium	5.5	mg/dL	–	–	–
Phosphate	3.4	mg/dL	–	–	–
Glucose	98	mg/dL	–	–	–
C‐reactive protein	1.73	mg/dL	–	–	–
HbA1c	5.3	%	–	–	–

**FIGURE 3:**
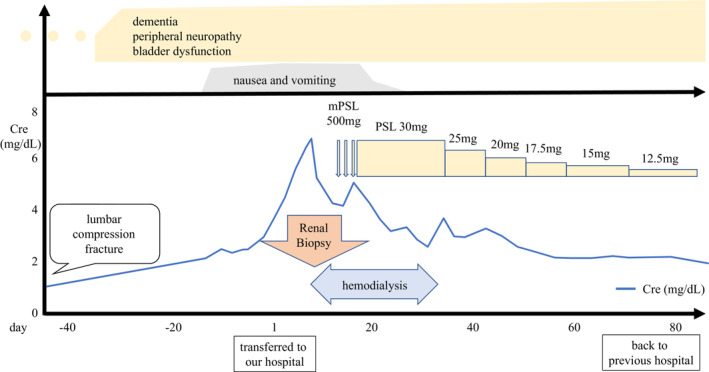
Clinical course of the patient.

The patient's clinical course is shown in Figure 3. Renal function deteriorated rapidly and temporary hemodialysis was initiated on Day 10. Confirming the renal biopsy findings on Day 15, intravenous methyl‐prednisolone pulse therapy (500 mg/day×3 days) followed by 30 mg/day of oral prednisolone was administered. Her vomiting showed signs of improvement since Day 21 and completely remitted by Day 37. Renal function improved and hemodialysis was withdrawn on day 39. The vomiting symptom resolved, but her dementia, peripheral neuropathy and bladder dysfunction gradually progressed. The serum creatinine level had improved to 2.13 mg/dL. However, persistent high‐grade proteinuria (8.2 g/gCr) still existed when the patient was transferred back to the previous hospital for rehabilitation on day 86.

## 3 DISCUSSION

The clinical spectrum of NIID has been broadened to include dementia, leukoencephalopathy, acute encephalitis‐like episodes, parkinsonism, essential tremor, peripheral neuropathy,^2^, ^3^, ^4^, ^5^, ^6^ and Liu et al. revealed that 55.9% of NIID patients had ever developed encephalitis‐like symptoms.^9^ In the present case, dementia, nausea and vomiting as an acute encephalitis‐like episode, peripheral neuropathy, and bladder dysfunction were consistent symptoms.

In addition, this patient suffered from rapid progressive glomerulonephritis. Renal biopsy revealed that only interstitial fibroblasts were positive for anti‐p62 immunofluorescence staining, indicating the presence of intranuclear inclusions. Other kidney‐specific cells such as tubular epithelial cells, podocytes or glomerular endothelial cells were not stained. In NIID patients, intranuclear inclusions are observed in neurons, glial cells and fibroblasts, which derive from neural crest cells in the process of development.^10^ Most fibroblasts in the kidney have similar developmental aspects because they also arise from neural crest cells.^11^ This insight explains why fibroblasts were exclusively positive for intranuclear inclusion in the renal biopsy specimen.

Generally, fibroblasts take various roles including extracellular matrix homeostasis, secretion of signaling factors for surrounding cells, regulation of tissue metabolism, as well as immune regulation and innate immunity.^12^ The multi‐hit pathogenesis of IgA nephropathy is proposed as follows. Abnormalities in the production of IgA1 lead to galactose‐deficient IgA1 (Gd‐IgA1) elevation, resulting in production of anti‐glycan antibodies via autoimmune response. Gd‐IgA1 and anti‐glycan antibodies form immune complexes and deposit in the glomerular mesangium. This deposition eventually activates the complement pathway and induce secretion of cytokines, chemokines and extracellular matrix proteins resulting in inflammation and fibrosis.^13^ Although it is difficult to prove the direct association between NIID and IgA nephropathy, dysfunction of fibroblasts with intranuclear inclusions may have played a partial role in this multi‐hit pathogenesis.

Previously, two reports referred to renal pathology of NIID patients. Horino et al. reported the case of mild mesangial proliferative glomerulonephritis with mesangial deposition of IgG, IgM, C3, and C1q, which was considered as mesangial proliferative glomerulonephritis‐like lupus nephritis, and oral prednisolone therapy was partly effective for reducing proteinuria.^14^ Motoki et al. reported that the renal biopsy specimen of a patient newly diagnosed as NIID which was performed 12 years before showed mesangial proliferative glomerulonephritis and was positive for intranuclear inclusions.^15^ However, these findings are inconsistent with those in our patient considering immunofluorescence findings and crescent formation. Huang et al. reviewed the possible mechanism of NIID. The length of GGC repeat expansion and the type and size of trinucleotide interruption can influence the subtype of NIID patient (dementia‐dominant, muscle‐weakness‐dominant, parkinsonism‐dominant), although the specific pathogenesis is unknown.^16^ NIID may influence the progression of renal diseases according to the style of repeat expansion.

In this case, digestive symptoms and kidney function were relieved after administration of steroid therapy, However, dementia, peripheral neuropathy, bladder disfunction, and massive proteinuria were resistant to steroid treatment. Although treatment for NIID has not been established,^17^ some case reports referred to the effectiveness of steroid therapy for the associated symptoms of NIID. Mori et al. reported that steroid pulse therapy followed by maintenance steroid treatment therapy improved consciousness disturbance,^18^ and Miyamoto et al. reported that high‐dose methyl‐prednisolone therapy was effective in terminating vomiting and abdominal pain in a pediatric NIID patient.^19^ The mechanism in the development of clinical manifestations of NIID include the direct genetic damage to the cells followed by the cell‐mediated immune response to the damage.^20^ Steroids could affect the immune response and this will be why steroids were partially effective to the patient.

In summary, to our knowledge, this is the first case of a NIID patient with crescentic IgA nephropathy successfully treated with steroid therapy. Although we could not provide a definite link between glomerulonephritis and NIID, further studies including case series study are needed to understand the pathogenesis of visceral organ dysfunction in NIID patients.

## AUTHOR CONTRIBUTIONS

**Keisuke Morita:** Writing – original draft. **Takahiro Shinzato:** Investigation. **Yuzo Endo:** Data curation. **Makoto Suzuki:** Investigation. **Hidefumi Yoshida:** Investigation. **Jun Sone:** Supervision. **Kojiro Nagai:** Supervision; writing – review and editing.

## FUNDING INFORMATION

No sources of funding were declared for this study.

## CONFLICT OF INTEREST STATEMENT

The authors state that they have no conflict of interest.

## CONSENT

Written informed consent was obtained from the patient for publication of this case report and accompanying images. A copy of the written consent is available for review by the Editor‐in‐Chief of this journal on request.

## Data Availability

The data are available from the corresponding author upon reasonable request.
